# Impact of renal function on the safety of biologic and synthetic DMARDs in rheumatoid arthritis: A retrospective cohort study from a Turkish tertiary center

**DOI:** 10.1097/MD.0000000000050387

**Published:** 2026-08-28

**Authors:** Dilara Bulut Gökten, Ömer Atakan Soğur, Zoljargal Sukhbaatar, Ridvan Mercan

**Affiliations:** a Division of Rheumatology, Department of Internal Medicine, Tekirdag Namik Kemal University, Tekirdag, Turkey; b Department of Internal Medicine, Tekirdag Namik Kemal University, Tekirdag, Turkey.

**Keywords:** chronic renal insufficiency, drug-related side effects and adverse reactions, rheumatoid arthritis, safety

## Abstract

This study aimed to evaluate treatment patterns, adverse events, and drug discontinuations associated with conventional synthetic (csDMARDs), biologic, and targeted synthetic disease-modifying antirheumatic drugs in patients with rheumatoid arthritis (RA) and chronic kidney disease (CKD). A retrospective cohort study was conducted on 264 RA patients with CKD, followed between 2015 and 2025. Renal function was assessed using estimated glomerular filtration rate (CKD-epidemiology collaboration) and categorized according to Kidney Disease Improving Global Outcomes guidelines into stages 3 to 5. Demographic data, laboratory parameters, comorbidities, medication history, and all drug-related adverse events were collected. Severe adverse events were defined as those requiring hospitalization, causing drug discontinuation, or resulting in death. Comparisons among CKD stages were performed using chi-square, Fisher exact test, analysis of variance, or Kruskal–Wallis tests as appropriate. Of 264 patients, the median age was 65 years, and 73.9% were female. Hypertension and diabetes were the leading comorbidities and major contributors to CKD etiology. Use of nephrotoxic csDMARDs (methotrexate, leflunomide) and nonsteroidal anti-inflammatory drugs (NSAIDs) decreased with advancing CKD, whereas glucocorticoid use increased. Toxicity from methotrexate, leflunomide, and NSAIDs rose significantly across renal stages (*P* < .05 for all). In contrast, adverse event rates for biologic disease-modifying antirheumatic drugs and targeted synthetic disease-modifying antirheumatic drugs did not differ significantly between CKD stages (all *P* > .05). No renal function-dependent increase in severe adverse events was observed for biologic or targeted therapies. In multivariable analysis, younger age, longer RA disease duration, higher baseline estimated glomerular filtration rate, and higher baseline Disease Activity Score 28-erythrocyte sedimentation rate were independently associated with the occurrence of any adverse event. While csDMARD and NSAID toxicity increase progressively with renal impairment, biologic and targeted synthetic agents maintain a stable safety profile across CKD stages.

## 1. Introduction

Rheumatoid arthritis (RA) is a chronic, systemic autoimmune disorder that primarily involves the synovial joints and may also present with various extra-articular manifestations. It is believed to arise from a complex interplay between genetic predisposition and environmental triggers. Based on the detection of immunoglobulin M rheumatoid factor and/or antibodies directed against citrullinated proteins, the disease is categorized as seropositive or seronegative.^[1]^ RA is one of the most common rheumatologic disorders, and its prevalence has been reported to range between 0.5% and 1%.^[2]^ In Turkey, the prevalence of RA has been reported to be approximately 0.56%.^[3]^ The main goals of treatment are to reduce inflammation, relieve pain, and prevent joint destruction. In current clinical practice, management typically involves the combined use of conventional synthetic (csDMARDs) and biologic disease-modifying antirheumatic drugs (bDMARDs).^[4]^

Chronic kidney disease (CKD) is a condition that, regardless of its underlying cause, has adverse effects on overall health. It is defined by the presence of structural kidney damage or abnormal renal function, typically evidenced by a persistently reduced estimated glomerular filtration rate (eGFR) below 60 mL/min/1.73 m^2^, lasting for at least 3 months.^[5]^ Although the exact prevalence is difficult to determine, CKD is estimated to occur in approximately one-fourth of patients with RA, which is considerably higher than that observed in the healthy population.^[6]^ During the course of RA, the causes of CKD are diverse; however, they can broadly be attributed to chronic inflammation and to drug-induced nephropathies, including nonsteroidal anti-inflammatory drugs (NSAIDs), methotrexate (MTX), and cyclosporine.^[7]^ Some studies have suggested that the disease itself, as well as inadequate disease control, may act as independent risk factors contributing to a decline in the eGFR.^[8]^

RA and CKD are interrelated conditions. While medications used in the treatment of RA may influence renal function, the presence of CKD can also affect the selection of drugs used in RA management.^[9]^ Since the metabolism and excretion of drugs used in the treatment of RA occur through renal pathways, alternative therapeutic options may need to be considered in patients with impaired kidneys. For instance, the use of MTX is often restricted due to CKD, and NSAID use requires particular caution in this patient group.^[10]^ Therefore, several studies have suggested that bDMARDs may serve as a cornerstone in these patients.^[11]^ In patients with RA and CKD, the available evidence regarding the use of bDMARDs is limited, consisting mainly of case reports and small case series.^[12]^ However, to the best of our knowledge, there are only a few studies that have evaluated bDMARDs, targeted synthetic DMARDs (tsDMARDs), and csDMARDs simultaneously in a patient population. The present study aimed to investigate all antirheumatic drugs used in RA, including biologic agents, in terms of treatment preferences, adverse events, and drug discontinuations due to adverse events, with particular consideration of CKD stages as an influencing factor.

## 2. Materials and methods

### 2.1. Data collection and exclusion criteria

This retrospective study included 264 patients with RA and CKD who were followed in the rheumatology outpatient clinic of a university hospital between 2015 and 2025. All patients fulfilled the 2010 American College of Rheumatology/European League Against Rheumatism classification criteria for RA.^[13]^ Renal function in patients was evaluated based on eGFR calculated using the CKD-epidemiology collaboration equation. In accordance with Kidney Disease Improving Global Outcomes guidelines, renal failure was categorized into 3 stages: stage 3 (eGFR = 30–59 mL/min/1.73 m^2^), stage 4 (eGFR = 15–29 mL/min/1.73 m^2^), and stage 5 (eGFR < 15).^[14]^ The exclusion criteria comprised the presence of coexisting rheumatic diseases, pregnancy or lactation, advanced hepatic failure, active malignancy, age under 18 years, severe heart failure, and active infections. Demographic information and laboratory findings were retrieved. Laboratory data included rheumatoid factor, anti-cyclic citrullinated peptide antibody, erythrocyte sedimentation rate (ESR), C-reactive protein, serum creatinine, and eGFR at baseline and at the 3-month follow-up. Disease activity was evaluated using tender joint counts, patient global assessment scores, and Disease Activity Score 28 (DAS28) status. No formal sample size calculation was performed due to the retrospective observational design of the study. All eligible patients meeting the inclusion criteria between 2015 and 2025 were included. Patients with incomplete clinical or laboratory data were excluded from the final analysis, and no imputation method was applied for missing data. The study covered a 10-year observation period.

Information regarding current and previous RA treatments, including DMARDs, glucocorticoids (GCs), and NSAIDs, was obtained. Because of the retrospective design, complete and standardized information regarding cumulative treatment duration for each medication was not consistently available across all patients. NSAID and MTX exposure in patients with advanced CKD largely reflected prior or ongoing treatments initiated before renal function deterioration, as well as therapies prescribed at external centers before referral. Comorbidities and the underlying causes of CKD were extracted from the hospital’s electronic medical records. Each adverse event was recorded, and those resulting in death, hospitalization, and/or treatment discontinuation were classified as severe. In patients receiving combination therapy, adverse events were attributed to the medication considered most likely responsible based on the treating rheumatologist’s documented clinical assessment in the electronic medical records. Attribution was supported by the temporal relationship between treatment exposure and the adverse event, improvement following drug withdrawal when documented, the known adverse-effect profile of the medication, and the absence of a more likely alternative explanation. Each adverse event was assigned to a single medication and was not counted repeatedly across concomitant therapies.

For the patient-level regression analysis, a composite binary outcome termed “any adverse event” was generated from the medication-specific adverse-event variables. Patients were classified as having an adverse event if at least 1 documented adverse event was recorded for any csDMARD, bDMARD, or tsDMARD, NSAID, or GC during the study period. Treatment inefficacy without a documented adverse event was not included in the composite outcome.

The study was approved by the Tekirdag Namik Kemal University Faculty of Medicine Non-Interventional Clinical Research Ethics Committee and conducted in accordance with the Declaration of Helsinki (approval number: 2025.36.02.14 and approval date: February 25, 2025). Written informed consent was obtained from all participants or their first-degree relatives.

### 2.2. Statistical analysis

All statistical analyses were performed using IBM SPSS Statistics version 28.0 (IBM Corp.). Continuous variables are presented as median (interquartile range [IQR]), as most variables were not normally distributed. The Shapiro–Wilk test and histogram inspection were used to assess normality. Categorical variables were presented as numbers and percentages. Comparisons between CKD stage 3, stage 4, and stage 5 were performed for each medication. The Pearson chi-square (χ^2^) test was used to compare categorical data such as the presence of total or severe adverse events. When the expected frequency in any cell was <5, the Fisher exact test was applied. For continuous parameters, differences between stages were evaluated by the Kruskal–Wallis test or one-way analysis of variance. A *P* value < .05 was considered statistically significant.

A multivariable binary logistic regression analysis was performed to identify patient-level factors associated with the occurrence of any adverse event. The dependent variable was the presence of at least 1 documented medication-related adverse event during the study period. Medication exposure variables were not included as predictors because the composite outcome was derived from medication-specific adverse-event records, and their simultaneous inclusion could introduce structural dependency between the predictors and the outcome. Age, RA disease duration, baseline eGFR, diabetes mellitus, hypertension, and baseline DAS28-ESR were selected a priori based on their established clinical relevance and potential association with treatment-related adverse events. Adjusted odds ratios (ORs) were reported with 95% confidence intervals (CIs). Model fit was evaluated using the Hosmer–Lemeshow goodness-of-fit test, Cox–Snell and Nagelkerke pseudo-*R^2^* values, and the area under the receiver operating characteristic curve. Baseline DAS28-ESR was calculated using the 28-joint tender and swollen joint counts, ESR, and patient global assessment.

## 3. Results

### 3.1. Demographic, laboratory, and clinical data of patients

A total of 264 patients with RA and CKD were included. The median age of the cohort was 65 years (IQR = 58–71), and 73.9% were female. The median disease duration was 8.0 years (IQR = 4.0–15.0), and the median CKD duration was 4.0 years (IQR = 2.0–6.0). Hypertension (39.7%) and diabetes mellitus (33.3%) were the most frequent comorbidities. Among patients with CKD, the most frequent etiologic factor was hypertension, accounting for 112 (42.4%), followed by diabetes mellitus in 54 patients (20.5%). Other identified causes included NSAID-associated nephropathy in 44 (16.7%), unspecified causes in 44 (16.7%), nephrolithiasis in 5 (1.9%), and glomerulonephritis in 2 (0.8%). The etiology remained uncertain in 3 patients (1.1%). Median baseline GFR was 36.0 mL/min/1.73 m^2^, with median serum creatinine of 1.8 mg/dL. Among stage 5 CKD patients (n = 82), 36 (43.9%) were receiving renal replacement therapy. Of these, 29 (80.6%) were on hemodialysis and 7 (19.4%) on peritoneal dialysis. The overall renal replacement therapy rate in the entire cohort was 13.6% (Table 1).

**Table 1:** Demographic, laboratory data, Disease Activity Scores, and comorbidities of patients.

Variables	RA and CKD Median (IQR) N = 264
Age (yr)	65.0 (58.0–71.0)
Sex (female), n (%)	195 (73.9)
Disease duration (yr)	8.0 (4.0–15.0)
CKD duration (yr)	4.00 (2.00–6.00)
Initial ESR (mm/h)	44.00 (26.00–70.50)
Initial CRP (mg/L)	9.00 (4.00–28.00)
Initial creatinine (mg/dL)	1.8 (0.9–4.2)
Initial GFR (mL/min/1.73 m^2^)	36.00 (12.00–60.00)
High global disease activity according to DAS28, n (%)	158 (59.8)
Initial patient global assessment (mm)	70.00 (60.00–80.00)
Anti-CCP positivity, n (%)	178 (67.4)
RF positivity, n (%)	189 (71.6)
Comorbidities, n (%)	
Hypertension	105 (39.7)
Diabetes mellitus	88 (33.3)
Hyperlipidemia	80 (30.6)
Coronary artery disease	80 (30.6)

CCP = cyclic citrullinated peptide, CKD = chronic kidney disease, CRP = C-reactive protein, DAS28 = Disease Activity Score 28, ESR = erythrocyte sedimentation rate, GFR = glomerular filtration rate, IQR = interquartile range, RA = rheumatoid arthritis, RF = rheumatoid factor.

### 3.2. Medical treatment use of patients

In the cohort, the majority were treated with systemic GCs (87.5%). Among csDMARDs, leflunomide (LEF; 67%) and hydroxychloroquine (HCQ; 64.3%) were the most commonly used, followed by sulfasalazine (SSZ; 25.7%) and MTX (17.4%). Across renal stages, GCs use increased with advancing CKD, from 84.7% in stage 3 to 97.5% in stage 5 (*P* = .003). In contrast, the administration of NSAIDs, MTX, and LEF declined as renal function worsened (*P* < .05 for each).

The adoption of bDMARD or tsDMARD remained relatively low overall. Among these, etanercept (ETN; 11.3%) and adalimumab (ADA; 7.5%) were the most frequently prescribed tumor necrosis factor (TNF) inhibitors, while rituximab (RTX; 8.3%) was the leading non-TNF biologic. Agents such as tocilizumab (TCZ), tofacitinib (TOF), and baricitinib (BAR) were used in fewer than 5% of cases, and abatacept (ABA) or upadacitinib (UPA) were rarely administered. None of the biologic or targeted drugs demonstrated a stage-dependent difference in use rate (*P* > .05; Table 2).

**Table 2:** Medical treatment use of patients.

	Stage 3 N = 92	Stage 4 N = 90	Stage 5 N = 82	Total N = 264	*P* value
Systemic glucocorticoid	78 (84.7)	73 (81.1)	80 (97.5)	231 (87.5)	***.003***
Intra-articular glucocorticoid	0 (0)	0 (0)	0 (0)	11 (4.17)	NA
NSAID	44 (47.8)	10 (11.1)	2 (2.4)	56 (21.2)	***<.001***
MTX	26 (28.2)	15 (16.6)	5 (6.1)	46 (17.4)	***.023***
Leflunomide	91 (98.9)	46 (51.1)	40 (48.7)	177 (67)	***<.001***
SSZ	24 (26.1)	22 (24.4)	22 (26.8)	68 (25.7)	.934
HCQ	61 (66.3)	58 (64.4)	51 (62.1)	170 (64.3)	.852
Prosthetic surgery	8 (8.6)	4 (4.4)	2 (2.4)	14 (5.3)	.167
Adalimumab	8 (8.6)	7 (7.7)	5 (6.1)	20 (7.5)	.808
Etanercept	12 (13.1)	10 (11.1)	8 (9.7)	30 (11.3)	.789
Infliximab	5 (5.4)	3 (3.3)	2 (2.4)	10 (3.8)	.564
Certolizumab pegol	3 (3.2)	2 (2.2)	3 (3.6)	8 (3.0)	.849
Golimumab	5 (5.4)	5 (5.5)	2 (2.4)	12 (4.5)	.544
Rituximab	9 (9.7)	9 (10)	4 (4.8)	22 (8.3)	.394
Tofacitinib	7 (7.6)	4 (4.4)	3 (3.6)	14 (5.3)	.461
Baricitinib	2 (2.1)	2 (2.2)	0 (0)	4 (1.5)	NA
Tocilizumab	4 (4.3)	5 (5.5)	1 (1.2)	10 (3.8)	.311
Upadacitinib	1 (1.1)	0 (0)	0 (0)	1 (0.3)	NA
Abatacept	2 (2.1)	1 (1.1)	0 (0)	3 (1.1)	NA

Bold and italic *P* values indicate statistically significant results (*P* < .05).

HCQ = hydroxychloroquine, MTX = methotrexate, NSAID = nonsteroidal anti-inflammatory drug, SSZ = sulfasalazine.

### 3.3. Most frequent adverse events and average medication doses

Considering the average drug doses and the most frequent adverse events, most patients treated with GCs (around two-thirds) received a low-to-moderate daily dose equivalent to ≤7.5 mg/d of prednisone, whereas the rest required higher doses. The most frequent complications were gastrointestinal (GIS) discomfort (12%) and elevated blood pressure (10%). For HCQ, the mean daily dose was approximately 250 mg/d. The most frequent adverse events included ocular irritation or early retinal changes (3%), allergic rash (3%), and mild GIS discomfort (2%). For MTX, mean weekly dose was 9.5 ± 4.2 mg, administered as 10 to 15 mg/wk in patients with stage 3 to 4 CKD. Adverse events were reported including elevated liver enzymes (28.8%), GIS intolerance (17.3%), and hematologic toxicity (9.6%). For LEF, daily dose was 17.2 ± 5.1 mg/d, most administered as 20 mg/d in 126 patients (71%). The most frequent adverse events were elevated liver enzyme levels (14%), hematologic toxicity (7%), and GIS intolerance (5%). For SSZ, the mean daily dose was 1.45 ± 0.52 g/d, most commonly 2000 mg/d in 35 patients (51%). The most frequent toxicities included elevated liver enzyme levels (9%) and allergic skin manifestations (5%). For NSAIDs, the mean daily dose, expressed in ibuprofen-equivalent terms, was approximately 1200 ± 450 mg/d. The most common adverse events were GIS irritation or bleeding (22%), hypertension and fluid retention (14%), and worsening renal function (12%).

Considering the average drug doses and the most frequent adverse events for biologics, for ADA, all patients received the drug at the standard subcutaneous dose of 40 mg every other week. The observed adverse events were mild injection site reactions in 2 patients, fatigue in 1 patient, and skin rash in another. For ETN, the drug was administered at the subcutaneous dose of 50 mg once weekly. Reported complications were injection site reactions (7%), upper respiratory tract infections (6%), and transient elevation of liver enzymes (5%). The patients received infliximab (IFX) therapy at a dose of 3 to 5 mg/kg intravenously every 8 weeks. The main adverse events were infusion-related reactions (20%), elevated liver enzymes (10%), and serious infections (10%). One patient (10%) developed tuberculous lymphadenitis, leading to immediate drug discontinuation. For RTX, each patient received 2 intravenous infusions of 1000 mg administered 2 weeks apart, repeated approximately every 6 months. The most common complications included mild infusion-related reactions (9%), transient lymphopenia (5%), and upper respiratory tract infections (5%). For TCZ, drug was administered intravenously at a dose of 8 mg/kg every 4 weeks, or subcutaneously at 162 mg once weekly. The frequent adverse events were elevated liver enzymes (20%), transient neutropenia (10%), and mild respiratory infections (10%). For certolizumab pegol (CZP) and golimumab (GOL), both agents were administered at standard subcutaneous doses (CZP: 400 mg at weeks 0, 2, 4, and then 200 mg every other week; GOL: 50 mg once monthly). The most common complications were injection site reactions, upper respiratory infections, and mild hepatic enzyme elevation. For Janus kinase (JAK) inhibitors, the most common complications included mild infections (8%), fatigue (6%), and transient elevations in liver enzymes (5%).

At the patient level, 220 of the 264 patients (83.3%) had at least 1 documented medication-related adverse event during the study period, whereas 44 patients (16.7%) had no recorded adverse event.

### 3.4. Comparison of adverse events among CKD stages

**Figure 1.**
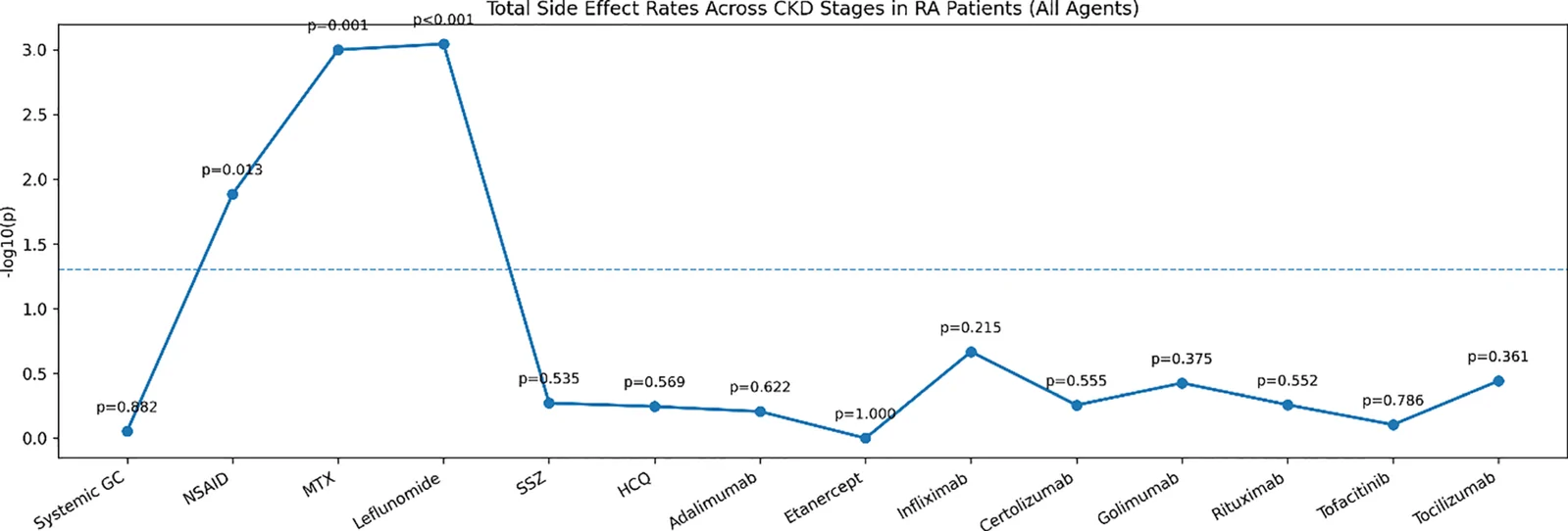
Total adverse event rates across CKD stages in RA patients according to medication groups. Total adverse event rates were compared across CKD stages (stage 3–5) in rheumatoid arthritis patients receiving different therapeutic agents. The y-axis displays −log10(p) values derived from chi-square tests, with the dashed red line indicating the threshold for statistical significance (*P* = .05). Among conventional therapies, NSAIDs, methotrexate, and leflunomide demonstrated a significant increase in total adverse event rates with advancing CKD stage (*P* = .013, *P* = .001, and *P* < .001, respectively). In contrast, no statistically significant differences in total adverse event rates were observed across CKD stages for biologic or targeted synthetic DMARDs (all *P* > .05). CKD = chronic kidney disease, DMARD = disease-modifying antirheumatic drug, GC = glucocorticoid, HCQ = hydroxychloroquine, MTX = methotrexate, NSAID = nonsteroidal anti-inflammatory drug, RA = rheumatoid arthritis, SSZ = sulfasalazine.

When adverse events were evaluated across renal failure stages, clear patterns emerged for several conventional agents. NSAIDs, MTX, and LEF were associated with a progressive rise in both total and severe side-effect frequencies as renal dysfunction advanced (*P* < .05 for all). For NSAIDs, total adverse events increased from 22.7% in stage 3 to 50% in stage 5 (*P* = .020), with severe reactions rising from 26.1% to 63.6% (*P* = .003). Likewise, MTX-related toxicity escalated from 22.7% to 32.1% (*P* = .013), and LEF from 15% to 40% (*P* < .001). Among bDMARD and tsDMARD, including TNF inhibitors (ADA, ETN, IFX, CZP, GOL), non-TNF biologics (RTX, TCZ, ABA), and JAK inhibitors (TOF, BAR, UPA), no statistically significant differences in adverse event incidence were observed across CKD stages (all *P* > .05). However, the number of patients receiving several individual biologic and targeted agents was limited, reducing the statistical power to detect potential between-stage differences (Table 3 and Fig. 1).

**Table 3:** Medications taken by RA patients according to the stages of renal failure and frequency of severe and total adverse events.

	Adverse events	Stage 3 N = 92	Stage 4 N = 90	Stage 5 N = 82	Total N = 264	*P* value	Most frequent events
Systemic glucocorticoid	Severe	9 (11.5)	7 (9.5)	9 (11.2)	25 (10.8)	.851	GIS discomfort, hypertension
	Total	26 (33.3)	24 (32.8)	29 (36.2)	79 (34.1)	.882	
Intra-articular glucocorticoid	Severe	0 (0)	0 (0)	0 (0)	0 (0)	NA	NA
	Total	0 (0)	0 (0)	0 (0)	0 (0)	NA	
NSAID	Severe	8 (18.1)	6 (60)	1 (50)	15 (26.7)	***.020***	GIS irritation/bleeding, hypertension/fluid retention, worsening renal function
	Total	10 (22.7)	7 (70)	1 (50)	18 (32.1)	***.013***	
MTX	Severe	9 (34.6)	9 (60.0)	4 (80.0)	22 (47.8)	***.006***	Elevated liver enzymes, GIS intolerance, hematologic toxicity
	Total	9 (34.6)	9 (60.0)	5 (100.0)	23 (50.0)	***.001***	
Leflunomide	Severe	14 (15.0)	19 (41.0)	12 (30)	45 (25.4)	***.003***	Elevated liver enzymes, leukopenia, GIS intolerance
	Total	14 (15.0)	23 (50.0)	16 (40.0)	53 (29.9)	***<.001***	
SSZ	Severe	6 (25.0)	2 (9.0)	5 (22.7)	13 (19.1)	.381	Elevated liver enzymes, allergic skin reactions
	Total	9 (37.5)	5 (22.7)	8 (36.3)	22 (32.3)	.535	
HCQ	Severe	3 (4.6)	5 (8.6)	6 (11.7)	14 (8.2)	.468	Ocular irritation/early retinal changes, allergic rash, GIS intolerance
	Total	5 (8.1)	5 (8.6)	7 (13.7)	15 (8.8)	.569	
Prosthetic surgery	Severe	1 (12.5)	1 (25)	1 (50)	3 (21.4)	.496	Infection, bleeding, re-surgery
	Total	1 (12.5)	2 (50)	1 (50)	4 (28.5)	.293	
Adalimumab	Severe	1 (12.5)	1 (14.2)	1 (20)	3 (15)	.928	Injection site reaction, fatigue, skin rash
	Total	1 (12.5)	2 (28.5)	1 (20)	4 (20)	.622	
Etanercept	Severe	1 (8.3)	1 (10)	2 (25)	4 (13.3)	.405	Injection site reaction, mild infection, transient liver enzyme rise
	Total	3 (25)	2 (20)	2 (25)	7 (23.3)	1.000	
Infliximab	Severe	1 (20)	1 (33.3)	2 (100)	4 (40)	.120	Infusion reactions, elevated liver enzymes, serious infection
	Total	2 (40)	1 (33.3)	2 (100)	5 (50)	.215	
Certolizumab pegol	Severe	1 (33.3)	1 (50)	1 (33.3)	3 (37.5)	1.000	Injection site reaction, upper respiratory infection, hepatic enzyme elevation
	Total	2 (66.6)	1 (50)	1 (33.3)	4 (50)	.555	
Golimumab	Severe	1 (20)	1 (20)	0 (0)	2 (16.6)	1.000	Injection site reaction, hepatic enzyme elevation, mild infection
	Total	2 (40)	3 (60)	0 (0)	5 (41.6)	.375	
Rituximab	Severe	1 (11.1)	1 (11.1)	0 (0)	2 (9)	1.000	Infusion reaction, lymphopenia, mild infection
	Total	2 (22.2)	3 (33.3)	0 (0)	5 (22.7)	.552	
Tofacitinib	Severe	2 (28.5)	1 (25)	1 (33.3)	4 (28.5)	1.000	Mild infection, fatigue, transient hepatic enzyme elevation
	Total	2 (28.5)	2 (50)	1 (33.3)	5 (35.7)	.786	
Baricitinib	Severe	0 (0)	1 (50)	0 (0)	1 (25)	NA	
	Total	0 (0)	1 (50)	0 (0)	1 (25)	NA	
Tocilizumab	Severe	2 (50)	1 (20)	1 (100)	4 (40)	.244	Elevated liver enzymes, neutropenia, respiratory infection
	Total	2 (50)	2 (40)	1 (100)	5 (50)	.361	
Upadacitinib	Severe	0 (0)	0 (0)	0 (0)	0 (0)	NA	NA
	Total	0 (0)	0 (0)	0 (0)	0 (0)	NA	
Abatacept	Severe	0 (0)	0 (0)	0 (0)	0 (0)	NA	NA
	Total	0 (0)	0 (0)	0 (0)	0 (0)	NA	

Bold and italic *P* values indicate statistically significant results (*P* < .05).

CKD = chronic kidney disease, GIS = gastrointestinal system, HCQ = hydroxychloroquine, MTX = methotrexate, NSAID = nonsteroidal anti-inflammatory drug, RA = rheumatoid arthritis, RRT = renal replacement therapy, SSZ = sulfasalazine.

In the multivariable logistic regression analysis, younger age was associated with a higher likelihood of having a documented adverse event (adjusted OR per 1-year increase = 0.94; 95% CI = 0.91–0.99; *P* = .008). Longer RA disease duration (adjusted OR per 1-year increase = 1.05; 95% CI = 1.01–1.10; *P* = .027), higher baseline eGFR (adjusted OR per 1 mL/min/1.73 m^2^ increase = 1.03; 95% CI = 1.00–1.05; *P* = .043), and higher baseline DAS28-ESR (adjusted OR per 1-point increase = 1.85; 95% CI = 1.08–3.17; *P* = .026) were independently associated with the occurrence of any adverse event. Diabetes mellitus and hypertension were not independently associated with the outcome. The overall model was statistically significant (likelihood-ratio χ^2^(6) = 24.94, *P* < .001) and demonstrated acceptable calibration according to the Hosmer–Lemeshow test (χ^2^(8) = 3.87, *P* = .869). The model had modest explanatory capacity (Cox–Snell *R^2^* = 0.090; Nagelkerke *R^2^* = 0.152) and moderate discrimination (area under the receiver operating characteristic curve = 0.732).

## 4. Discussion

The current investigation evaluated treatment patterns and drug-related adverse events of conventional, biologic, and tsDMARDs among patients with RA who also had CKD. The analysis focused on whether the degree of renal impairment influenced the safety of these therapies. Within this cohort, toxicity related to conventional drugs showed a notable increase as kidney function deteriorated, while biologic and targeted agents did not demonstrate a similar pattern. GCs use tended to rise in advanced CKD, whereas nephrotoxic conventional DMARDs were prescribed less frequently, indicating an adjustment in clinical decision-making according to renal capacity. Collectively, the results imply that kidney dysfunction may have limited impact on the safety of biologic therapies in RA, suggesting that these drugs may remain reasonable options for individuals with CKD when conventional treatments are contraindicated or poorly tolerated.

Because TNF inhibitors are primarily eliminated through intracellular proteolysis rather than renal excretion, their pharmacokinetics are largely unaffected by impaired renal function. This biological characteristic may explain the stable safety profile observed across CKD stages in the present cohort.^[15,16]^ Consistent with the findings of Yoshimura et al and previous reports demonstrating that biologic DMARDs can be used effectively and safely even in patients with advanced CKD or on dialysis,^[11]^ the present results indicate that there was no significant variation in the frequency or severity of adverse events related to TNF inhibitors across CKD stages, suggesting that renal impairment does not substantially modify the safety profile of biologic or tsDMARDs. While Yoshimura et al focused solely on first-line biologic agents, mainly TNF, interleukin-6 (IL-6), and cytotoxic T-lymphocyte-associated protein 4 inhibitors, and identified IL-6 blockade as the most durable option in individuals with eGFR < 30 mL/min/1.73 m^2^, the current cohort extends these observations by encompassing a broader therapeutic spectrum that includes csDMARDs, bDMARDs, and tsDMARDs. Unlike the cohort of Yoshimura et al, which did not include JAK inhibitors, the present study also evaluated tsDMARDs and demonstrated a similarly stable safety profile across CKD stages.

Consistent with current recommendations, MTX toxicity increased markedly with advancing CKD, reflecting reduced renal clearance.^[17]^ Similarly, LEF, which is not efficiently cleared by either hemodialysis or peritoneal dialysis,^[18]^ showed a higher frequency of hepatic and hematologic adverse events in advanced CKD stages, supporting the caution advised for moderate-to-severe renal impairment.^[19]^ In contrast, HCQ, which is primarily metabolized in the liver, remained well tolerated without dose-dependent toxicity, consistent with the observation by Tyczyńska et al that only prolonged therapy may require dose reduction in the presence of renal impairment.^[20]^ SSZ exhibited a mild toxicity profile in both works,^[20]^ with tolerable doses up to 1 g/d even in dialysis patients, although close monitoring was recommended.^[21]^

For tsDMARDs, the present cohort provides clinical data complementing Weiner et al’s pharmacokinetic summary^[22]^ and Patschan et al’s recent review.^[23]^ TOF and BAR were administered at standard or slightly reduced doses, yet no renal stage-dependent increase in adverse events was observed, suggesting that JAK inhibitors may be safely used with appropriate dose adjustment in severe CKD.^[24]^ UPA also demonstrated acceptable tolerability, consistent with prior pharmacologic guidance that cautions only in end-stage renal disease.^[25]^ In contrast, recent findings from Nakayama et al indicated that JAK inhibitors showed lower drug retention due to adverse events in patients with moderate-to-severe CKD, whereas bDMARDs, including TNF, IL-6, and cytotoxic T-lymphocyte-associated protein 4 inhibitors, maintained stable safety profiles across renal stages.^[26]^ Unlike this ANSWER study, which did not include csDMARDs, the present cohort also evaluated csDMARDs. Among biologic agents, findings again were concordant with the Speeckaert et al and conclusion that no formal dose modification is required for TNF inhibitors,^[27]^ RTX or ABA. In the current cohort, ADA, ETN, and IFX showed no significant variation in adverse events across renal stages, while TCZ remained well tolerated, consistent with the observations reported by Seneschall et al^[28]^ and Fukuda et al.^[29]^ Unlike the review by Patschan et al, which focused exclusively on end-stage renal disease and theoretical pharmacokinetic considerations,^[23]^ the present analysis provides real-world evidence supporting the safe use of biologic and tsDMARDs in patients with earlier CKD stages. Taken together, these drug-specific comparisons indicate that while csDMARDs demonstrate progressive nephrotoxicity, biologic and tsDMARDs maintain a stable safety profile across CKD stages when appropriately selected and monitored.

The patient-level multivariable analysis suggested that younger age, longer RA disease duration, higher baseline eGFR, and higher baseline DAS28-ESR were associated with the recording of at least 1 adverse event. The association with longer disease duration may reflect greater cumulative treatment exposure, although cumulative exposure could not be standardized in this retrospective study. The association between higher eGFR and adverse events should be interpreted cautiously. Patients with more advanced renal impairment may have been less likely to receive potentially nephrotoxic or poorly tolerated agents, or such treatments may have been discontinued earlier, resulting in treatment-selection and survivorship effects. Similarly, the inverse association with age should not be interpreted as evidence that older age protects against adverse events. Differences in treatment intensity, medication selection, follow-up, and documentation may have contributed to this finding. Although the model demonstrated acceptable calibration and moderate discrimination, its explanatory capacity was modest. This indicates that a substantial proportion of the variability in adverse-event occurrence was not captured by the available clinical variables. Unmeasured factors, including cumulative drug exposure, dose changes, polypharmacy, frailty, treatment adherence, and the temporal relationship between exposure and adverse events, may have influenced the findings.

This study has certain limitations that warrant consideration. The retrospective, single-center design may have introduced a degree of selection bias and restricts the generalizability of the results to other populations. To reduce this risk, all consecutive eligible patients followed during the study period were included. Due to the retrospective design and limitations of the institutional electronic medical record system, it was not possible to reliably identify all patients initially screened for eligibility. Consequently, the exact number of patients excluded because of incomplete clinical or laboratory data could not be reconstructed, representing a potential source of selection bias. Because adverse-event attribution was based on retrospective clinical documentation rather than standardized causality assessment tools, some degree of misclassification cannot be excluded, particularly in patients receiving combination therapy.

Laboratory variables reflecting renal status, such as proteinuria or urinary sediment analyses, were not consistently available, which limited a more comprehensive evaluation of renal outcomes. Additionally, because adverse events were identified retrospectively from electronic medical records, mild or self-limited adverse events that were not documented during routine clinical practice may have been underreported, potentially leading to an underestimation of the overall adverse event frequency.

The relatively small number of patients receiving several biologic and targeted synthetic DMARDs substantially limited statistical power for individual drug analyses. Consequently, the absence of statistically significant differences in adverse event rates across CKD stages should not be interpreted as evidence of equivalent safety for these therapies. Larger prospective studies with adequate representation of individual biologic and targeted agents are required before firm conclusions regarding comparative safety can be drawn. Although extensive data were obtained from institutional medical records, cumulative treatment exposure and the exact timing of adverse events could not be consistently standardized because of the retrospective design. Therefore, differences in treatment duration may have influenced the observed frequency of adverse events and should be considered when interpreting comparisons between treatment groups.

The composite adverse-event outcome was derived retrospectively from medication-specific adverse-event records and represented whether a patient had at least 1 recorded adverse event during the entire observation period. It therefore did not account for differences in treatment duration, cumulative exposure, repeated events, or time at risk. Moreover, the high frequency of the composite outcome and possible differences in clinical documentation should be considered when interpreting the regression estimates. Although medication exposure variables were deliberately excluded from the patient-level model to avoid structural dependency with the composite outcome, residual confounding by treatment selection remains possible.

Despite these constraints, the study provides valuable real-world evidence from a tertiary rheumatology cohort and contributes to understanding the safety of antirheumatic therapies across different stages of CKD. Larger, multicenter, prospective studies integrating pharmacokinetic data and long-term renal follow-up are needed to confirm these findings and to establish more precise therapeutic strategies for patients with RA and impaired kidney function.

## 5. Conclusion

In summary, the present study highlights that while csDMARDs show a progressive increase in toxicity with declining renal function, biologic and targeted synthetic agents showed no apparent increase in adverse events across CKD stages in this cohort. However, these findings should be interpreted cautiously because several treatment groups contained only a limited number of patients. Patient-level adverse-event occurrence was also associated with disease duration, baseline disease activity, age, and eGFR; however, these exploratory findings require cautious interpretation because of the retrospective design and unmeasured differences in cumulative treatment exposure. These findings emphasize the importance of individualized drug selection in patients with RA and impaired kidney function. Biologic therapies may represent a potentially safer alternative when conventional treatments are limited by nephrotoxicity or intolerance. Although further prospective and multicenter research is needed to validate these results, the current real-world data provide practical guidance for optimizing treatment strategies in this complex clinical setting.

## Acknowledgments

The authors would like to thank Dr Mehmet Engin Tezcan for his valuable assistance with the statistical analyses.

## Author contributions

**Conceptualization:** Dilara Bulut Gökten, Ridvan Mercan.

**Data curation:** Dilara Bulut Gökten, Ömer Atakan Soğur, Zoljargal Sukhbaatar.

**Formal analysis:** Dilara Bulut Gökten.

**Investigation:** Dilara Bulut Gökten, Ömer Atakan Soğur, Zoljargal Sukhbaatar.

**Methodology:** Dilara Bulut Gökten, Ömer Atakan Soğur.

**Resources:** Dilara Bulut Gökten.

**Validation:** Dilara Bulut Gökten, Ridvan Mercan.

**Supervision:** Ridvan Mercan.

**Visualization:** Ridvan Mercan.

**Writing – original draft:** Dilara Bulut Gökten, Ridvan Mercan.
